# Thymoquinone: An IRAK1 inhibitor with *in vivo* and *in vitro* anti-inflammatory activities

**DOI:** 10.1038/srep42995

**Published:** 2017-02-20

**Authors:** Muhammad Jahangir Hossen, Woo Seok Yang, Daewon Kim, Adithan Aravinthan, Jong-Hoon Kim, Jae Youl Cho

**Affiliations:** 1 Department of Genetic Engineering, Sungkyunkwan University, Suwon 16419, Republic of Korea; 2 Department of Animal Science, Patuakhali Science and Technology University, Dumki, Patuakhali 8602, Bangladesh; 3 Laboratory of Bio-informatics, Department of Multimedia Engineering, Dankook University, Cheonan 31116, Republic of Korea; 4 Department of Physiology, College of Veterinary Medicine, Chonbuk National University, Iksan 54596, Republic of Korea

## Abstract

Thymoquinone (TQ) is a bioactive component of black seed (*Nigella sativa*) volatile oil and has been shown to have anti-oxidative, anti-inflammatory, and anti-cancer properties. In the present study, we explored the molecular mechanisms that underlie the anti-inflammatory effect of TQ and its target proteins using lipopolysaccharide (LPS)-stimulated murine macrophage-like RAW264.7 and human monocyte-like U937 cells, together with LPS/D-galactosamine (GalN)-induced acute hepatitis and HCl/EtOH-induced gastritis mouse models. TQ strongly inhibited the production of nitric oxide (NO) and repressed NO synthase (iNOS), tumor necrosis factor (TNF)-α, cyclooxygenase (COX)−2, interleukin (IL)−6, and IL-1β expression in LPS-activated RAW264.7 cells. Treatment of LPS/D-GalN–induced hepatitis and EtOH/HCl–induced gastritis mouse models with TQ significantly ameliorated disease symptoms. Using luciferase reporter gene assays, we also showed that the nuclear levels of transcription factors and phosphorylation patterns of signaling proteins, activator protein (AP)−1, and nuclear factor (NF)-κB pathways were all affected by TQ treatment. Finally, we used additional kinase and luciferase validation assays with interleukin-1 receptor-associated kinase 1 (IRAK1) to show that IRAK1 is directly suppressed by TQ treatment. Together, these findings strongly suggest that the anti-inflammatory actions of TQ are caused by suppression of IRAK-linked AP-1/NF-κB pathways.

Inflammation is an important component of the physiological response to harmful stimuli such as infection^1^. Acute and prolonged inflammatory events perform essential roles in promoting innate immunity, maintaining immune system homeostasis, and as a natural defense mechanism^2^. Most studies to date have aimed to understand the pathophysiological mechanisms of the inflammatory response^3^. However, chronically sustained inflammation can cause serious diseases including cancer, Alzheimer’s disease, arthritis, asthma, hypertension, and atherosclerosis^4^,^5^. Therefore, preventing the episodic upregulation of the inflammatory response could provide a way to prevent many serious diseases^6^.

Toll-like receptors (TLRs) recognize pathogen-derived molecules such as lipopolysaccharides (LPS), pam3csk, and poly (I:C), leading to activation of immune cells including dendritic cells and macrophages and ultimately triggering an inflammatory response^7^. This inflammatory response includes the induction of intracellular signaling that involves inhibition of tyrosine kinases (Src, Syk, JAK, and interleukin receptor-associated kinase [IRAK1]), AGC protein kinase, and κB kinase (IKK), which in turn activate nuclear transcription factor kappa B (NF-κB) and induce the gene expression of numerous inflammation-regulatory proteins such as cyclooxygenase (COX)−2, lipoxygenase, and inducible nitric oxide synthase (iNOS)^8^,^9^,^10^,^11^. Subsequently, a variety of inflammatory mediators including leukotrienes, histamines, prostaglandin E_2_ (PGE_2_), nitric oxide (NO), cytokines, and chemokines are released and further promote the chemotactic response in neighboring inflammatory cells, inducing the biosynthesis of hydrolytic enzymes such as matrix metalloproteinases^12^,^13^. Although all of these reactions induce resistance against pathogenic infection, chronically prolonged inflammatory events can result in serious diseases. Consequently, immunologists have begun to focus on developing safe and effective anti-inflammatory treatments that alleviate prolonged inflammatory symptoms.

Interleukin receptor-associated kinase is a serine/threonine kinase that regulates various signaling events from Toll-like receptors (TLRs) to transcription factors, including nuclear factor (NF)-κB and activator protein (AP)−1, in inflammatory cells such as macrophages^14^. Activation of IRAK1 requires its phosphorylation on threonine 209; this triggers recruitment of TRAF6, leading to the activation of transforming growth factor β-activated kinase 1 (TAK1) and mitogen-activated protein kinase (MAPK)^15^. The key role of IRAK1 in inflammatory responses has been demonstrated in several inflammatory disease models such as high-fat diet–induced non-alcoholic steatohepatitis^16^, endotoxic shock^17^, LPS-induced acute lung injury^18^, lupus nephritis^19^, and liver ischemia/reperfusion injury^20^. Similarly, IRAK1/4 inhibitory compounds including mangiferin, kalopanaxsaponin B, anthraquinone-2-carboxylic acid, and caffeic acid were reported to exhibit curative activities against inflammatory diseases such as 2,3,4-trinitrobenzene sulfonic acid–induced colitis^21^,^22^,^23^,^24^. Strong and selective inhibition of IRAK1 is therefore considered an effective approach for treating inflammatory diseases.

Thymoquinone (TQ, Fig. 1a) is the major (30–48%) bioactive component of black seed (*Nigella sativa*, Ranunculaceae family) volatile oil^25^,^26^. Previous work has shown that TQ has anti-oxidative, anti-inflammatory, and anti-cancer activities. Specifically, TQ has shown efficacy in alleviating symptoms in several disease models, including cancer, diabetes, asthma, encephalomyelitis, and arthritis^27^. It has been suggested that TQ acts as a radical scavenger and thereby preserves the enzymatic activity of antioxidants like catalase, glutathione peroxidase, and glutathione-*S*-transferase^28^. The anti-cancer effects of TQ have been reported to be mediated by various modes of action, including cell growth arrest, pro-apoptosis or anti-proliferative functions, reactive oxygen species (ROS) release, and anti-metastasis/angiogenesis activity^29^,^30^. The reported anti-oxidative and anti-inflammatory effects of this molecule have led to multiple studies seeking to characterize its molecular mechanisms and to assess its potential use in treating inflammatory diseases^27^. Despite numerous pharmacological studies, however, the precise molecular mechanisms by which TQ induces anti-inflammatory effects have not been fully elucidated. The work presented here seeks to uncover these molecular mechanisms and to identify the target proteins of TQ *in vitro* using LPS-activated macrophages and *in vivo* using LPS/D-GalN–induced hepatitis and EtOH/HCl–induced gastritis mouse models, together with data collected from luciferase reporter gene and kinase assays, an overexpression strategy, and immunoprecipitation analysis.

## Results

### Effect of TQ on *in vitro* inflammatory responses

**Figure 1:**
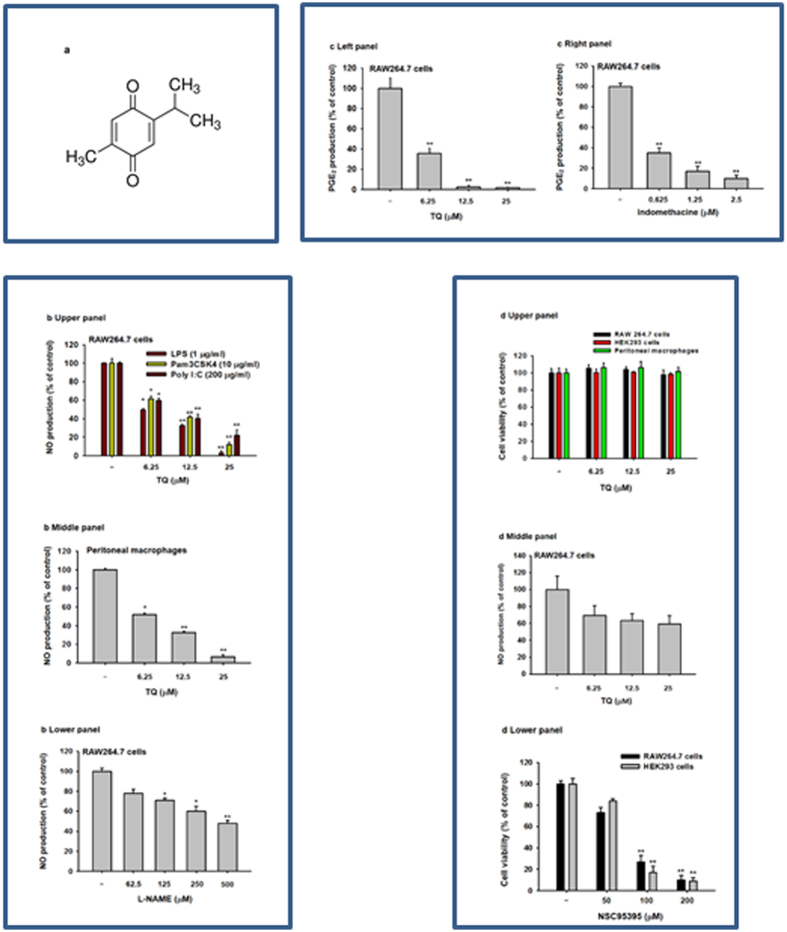
Effect of TQ on *in vitro* inflammatory responses. (**a**) Chemical structure of TQ. (**b** and **d** middle panel) Supernatant NO levels of RAW264.7 cell cultures (upper panel) and peritoneal macrophages (middle panel) treated with or without LPS (1 μg/mL), pam3CSK (10 μg/mL), or poly (I:C) (200 μg/mL) in the presence or absence of TQ or L-NAME (lower panel) were analyzed using the Griess assay. (**c**) Supernatant PGE_2_ levels in RAW264.7 cell cultures treated with LPS (1 μg/mL) in the presence or absence of TQ (left panel) or indomethacine (right panel) were analyzed by EIA. (**d**) Cell viability of RAW264.7 cells, HEK293 cells, and peritoneal macrophages treated with TQ (upper panel) or NSC95395 (lower panel) for 24 h was determined using the MTT assay. Data (**b** to **d**) shown represent mean ± SD of five samples. *p < 0.05 and **p < 0.01 compared with control.

To evaluate the potential effects of TQ on inflammatory responses, we initially quantified its effect on the secretion of NO and PGE_2_ in LPS-stimulated macrophage-like RAW264.7 cells. At a concentration of 25 μM, TQ strikingly (p < 0.01) suppressed LPS-, pam3CSK-, and Poly(I:C)-mediated NO production in a dose-dependent manner by up to 97% in activated RAW264.7 cells (Fig. 1b, upper panel) and by 95% in peritoneal primary macrophages (Fig. 1b, middle panel). Under the same conditions, TQ also effectively (p < 0.01) decreased LPS-triggered PGE_2_ release by up to 99% at 25 μM (Fig. 1c, left panel). We also showed that the standard compounds L-NAME (Fig. 1b, lower panel) and indomethacine (Fig. 1c, right panel) dose-dependently reduced the secretion of NO and PGE_2_ under the same conditions. TQ treatment maintained intact viability of RAW264.7 cells, primary macrophages, and HEK293 cells (Fig. 1d, upper panel) at the concentrations that suppressed NO and PGE_2_ release (Fig 1b and c), in contrast to NSC95395 (2,3-bis-[(2-hydroxyethyl)thio]-1,4-naphthoquinone) (Fig. 1d, lower panel). This suggests that the NO and PGE_2_ inhibitory effects of TQ are not due to non-specific toxicity. Meanwhile, there was no remarkable inhibition of NO production under TQ treatment conditions without LPS stimulation (Fig. 1d, middle panel).

### Effect of TQ on *in vivo* inflammatory responses

**Figure 2:**
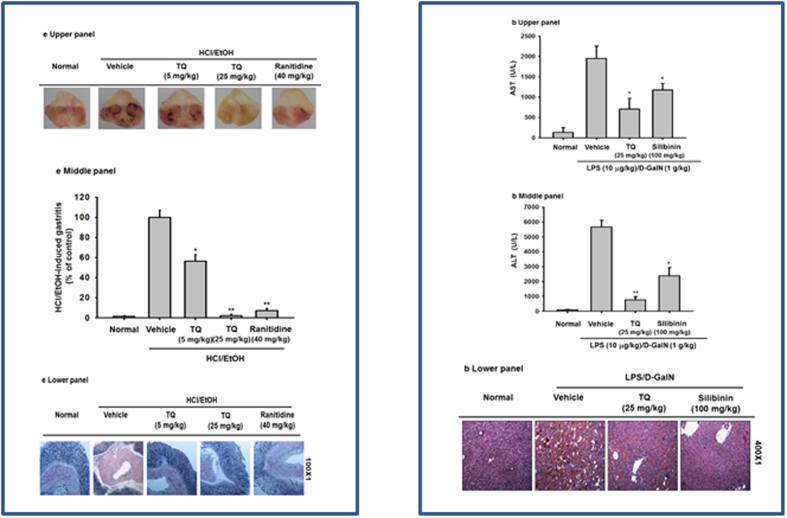
Effect of TQ on *in vivo* inflammatory responses. (**a**) Mice were orally treated with TQ (5 and 25 mg/kg) or ranitidine (40 mg/kg) for 3 days before induction of gastritis with HCl/EtOH. After 1 h, the gastric lesions were measured using ImageJ (middle panel), photography (upper panel), and histopathological examination (lower panel). (**b**) Mice were orally treated with TQ (25 mg/kg) or silibinin (100 mg/kg) for 6 days before intraperitoneal LPS/D-GalN injection. After 1 h, mice were sacrificed, and serum was prepared for biochemical parameter analysis of AST (upper panel) and ALT (middle panel) levels, and liver tissue was stained for histopathological examination (lower panel). Data (**b** to **d**) shown represent the mean ± SD of seven mice. *p < 0.05 and **p < 0.01 compared with control.

We further investigated the *in vivo* clinical anti-inflammatory capabilities of TQ using an HCl/EtOH-induced gastritis mouse model. Oral administration of TQ (at 5 and 25 mg/kg) significantly reduced (p < 0.01) gastritis by up to 98%, an effect comparable to the 97% reduction seen with ranitidine (40 mg/kg), a standard drug with anti-ulcer activity (Fig. 2a, upper and middle panels). Hematoxylin and eosin (H&E) staining of stomach tissue showed that inflammation in HCl/EtOH–induced gastritis involves the recruitment of abundant neutrophils, and pretreatment with TQ prevents this immune cell recruitment (Fig. 2a, lower panel), potentially explaining its strong anti-gastritis effect. We also tested the potential of TQ as a protective agent against liver damage induced by LPS/D-GalN using a hepatitis animal model. Oral treatment with TQ (25 mg/kg) significantly reduced the initially high levels of ALT (6,306 U/L) and AST (6,661 U/L) induced by LPS by up to 75 and 90%, respectively, similar to the effect of silibinin, a flavonolignan compound extracted from the fruits and seeds of *Silybum marianum*^31^ and with reported anti-hepatitis activity (Fig. 2b, upper and middle panels). Additionally, H&E staining of hepatic tissue showed that LPS/D-GalN–induced hepatic inflammation causes significant neutrophilic recruitment, which was prevented by pretreatment with TQ and silibinin (Fig. 2b, lower panel). Together, these findings suggest that TQ exerts a substantial hepatoprotective effect.

### Effect of TQ on the transcription regulatory functions of NF-κB and AP-1

**Figure 3:**
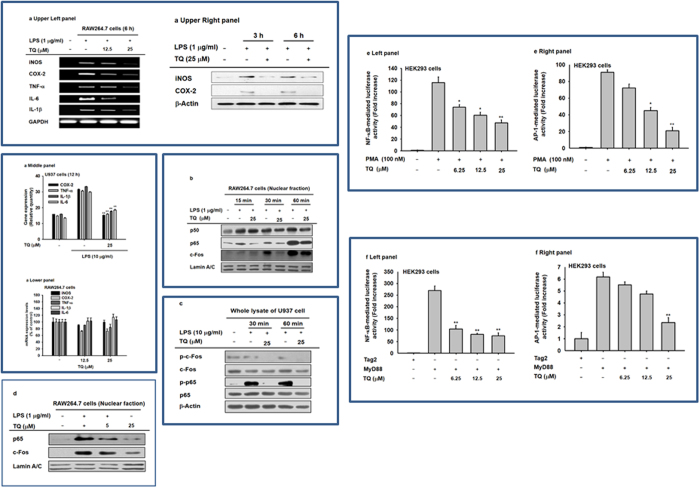
Effects of TQ on the transcriptional activation of NF-κB– and AP-1–driven reporter construct and the expression of pro-inflammatory genes. (**a**) The mRNA levels of iNOS and COX-2, TNF-α, IL-6, and IL-1β were determined by semiquantitative RT-PCR in LPS-treated RAW264.7 cells (upper left panel) and RAW264.7 cells (lower panel) or by real-time RT-PCR using mRNA from LPS/PMA-treated U937 cells (middle panel) during TQ exposure (**a**, upper right panel). Levels of iNOS, COX-2, and β-actin in whole cell lysates of LPS-treated RAW264.7 cells pretreated with TQ (25 μM) were determined by immunoblot analysis. (**b**) Nuclear translocation of p65, p50, c-Fos, and lamin A/C was determined by immunoblot analysis. (**c**) Phosphorylated and total protein levels of c-Fos, p65, and β-actin in whole cell lysates were determined by immunoblot analysis of LPS/PMA-treated U937 cells. (**d**) Levels of p65, c-Fos, and lamin A/C in nuclear fractions of TQ-pretreated RAW264.7 cells stimulated with LPS for 1 h were determined by immunoblot analysis. (**e** and **f**) HEK293 cells were transfected with 1 μg/mL NF-κB– (left panel) or AP-1– (right panel) Luc and β-gal (as a transfection control) plasmids. HEK293 cells were treated with PMA (100 nM) or cotransfected with a MyD88 construct (1 μg/mL). Luciferase activity was measured using a luminometer. The gel and blots in **b**, **c**, and **d** were run under the same experimental conditions and are shown as cropped gels/blots (original gels/blots with indicated cropping lines are shown in Supplementary Figure S1). Results (**a** to **d**) are shown for one representative experiment of three. Data (**e** to **f**) shown represent mean ± SD of five samples. *p < 0.05 and **p < 0.01 compared with control.

To study whether the TQ-driven suppression of NO and PGE_2_ production is modulated at the transcriptional or translational stages, the mRNA levels of inflammatory genes regulating NO and PGE_2_ release were examined. Using RT-PCR, we showed that LPS treatment significantly upregulated iNOS, COX-2, TNF-α, IL-1β, and IL-6 mRNA expression in macrophage-like RAW264.7 cells (Fig. 3a, upper left panel). Similar to the effect of TQ on mRNA, this compound also reduced the protein expression of iNOS and COX-2, as detected by immunoblotting analysis (Fig. 3a, upper right panel). We also showed upregulation of COX-2, TNF-α, IL-1β, and IL-6 mRNA levels in PMA/LPS-treated U937 cells (Fig. 3a, middle panel). This gene upregulation was reversed by TQ treatment (25 μM); however, TQ alone did not affect the mRNA levels of inflammatory genes (iNOS, COX-2, TNF-α, IL-1β, and IL-6) under normal culture conditions (Fig. 3a, lower panel). Moreover, TQ successfully blocked the LPS-driven translocation of p65 (a subunit of NF-κB) into the nucleus of LPS-stimulated RAW264.7 cells between 15 and 60 min after treatment (Fig. 3b). In addition, TQ reduced the expression of phosphorylated forms of c-Jun and p65 between 30 and 60 min after application in PMA/LPS-treated U937 cells (Fig. 3c). Dose-dependent inhibition of p65 and c-Fos translocation by TQ (5 and 25 μM) was confirmed in LPS-treated RAW264.7 cells (Fig. 3d). We also examined whether TQ suppressed the activation and translocation of NF-κB and AP-1 using luciferase expression reporter constructs with AP-1– or NF-κB–binding sites transfected into PMA-treated or MyD88-transfected HEK293 cells. Previous studies confirmed that luciferase activity of AP-1- and NF-κB was strongly upregulated in these systems^32^,^33^. Consistent with our previous findings, TQ dose-dependently inhibited luciferase activity driven by these genes by up to 60–85% at a concentration of 25 μM (Fig. 3e and f).

### Effect of TQ on signaling events upstream of NF-κB and AP-1 activation

**Figure 4:**
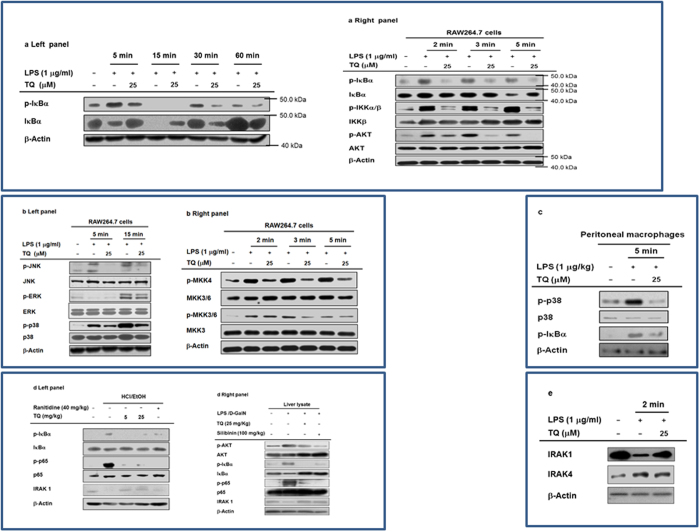
Effect of TQ on the activation of upstream signaling enzymes in NF-κB and AP-1 translocation. (**a**,**b**,**c** and **d**) Phosphorylated and total protein levels of IκBα, IKKα/β, AKT, PDK1, ERK, JNK, p38, MKK3, MKK3/6, MKK4, IRAK1, IRAK4, and β-actin from whole cell or tissue lysates of LPS-treated RAW264.7 cells (**a**,**b** and **e**), peritoneal macrophages (**c**), stomach (d left panel), and liver (d right panel) were determined by immunoblot analysis. The blots in **a**, **b**, **c**, **d**, and **e** were run under the same experimental conditions and are shown as cropped blots (original blots with indicated cropping lines are shown in Supplementary Figure S1). Results are shown for one representative experiment of three.

To explore the suppressive action of TQ on intracellular signaling components involved in activation of NF-κB or AP-1, we measured the phosphorylation levels of relevant signaling molecules. We found that TQ successfully suppressed the LPS-mediated increase in phosphorylated IκBαα at 5, 30, and 60 min after treatment with LPS (Fig. 4a, left panel). TQ also strongly inhibited the phosphorylation of IκBα, IKKα/β, AKT, and PDK1 at 2 and 5 min after LPS treatment (Fig. 4a, right panel), implying that the upstream regulators of these enzymes are relevant molecular targets of TQ.

Next, we used immunoblot analysis to determine whether TQ inhibits MAPK phosphorylation, which is important for regulating downstream targets that mediate the inflammatory effects of LPS^34^. We found that TQ blocked the induction of ERK, JNK, and p38 MAPK phosphorylation at 5 min (Fig. 4b, left panel). LPS treatment increased phosphorylation of MEK1/2, MKK4, and MKK3/6, which are upstream of ERK, JNK, and p38, respectively; TQ suppressed all of these upstream mediators 2, 3, and 5 min after LPS treatment (Fig. 4b, right panel). This suggests that TQ affects one of the signaling cascades upstream of ERK, JNK, and p38 MAPK activation in its anti-inflammatory activity. Similar to these findings, TQ (25 μM) inhibited the LPS-mediated upregulation of both phospho-p38 and phospho-IκBα in peritoneal macrophages 5 min after treatment with LPS (Fig. 4c), implying that primary macrophages show a similar inhibitory pattern to RAW264.7 cells. Further supporting these findings, we showed that oral treatment with TQ blocked the increased IκBα and p65 phosphorylation in the stomach of HCl/EtOH-treated mice (Fig. 4d, left panel) and also reduced the phosphorylation of MKK4 and c-Jun (components of the MAPK/AP-1 pathway) and p65, IκBα, and AKT (components of the NF-κB pathway) in the LPS/D-GalN-induced hepatitis mouse model (Fig. 4d, right panel). Finally, we tested the inhibitory activity of TQ on IRAKs. Interestingly, both HCl/EtOH and LPS/D-GalN remarkably decreased the protein level of IRAK1, whereas TQ treatment clearly restored the decreased level to a normal state (Fig. 4d). Moreover, TQ inhibited the decrease of IRAK1 at 2 min after LPS treatment (Fig. 4e), in stomach of HCl/EtOH-treated mice (Fig. 4d, left panel), and in liver of LPS/D-GalN-exposed mice (Fig. 4d, right panel).

### Effect of TQ on IRAK1 activation

**Figure 5:**
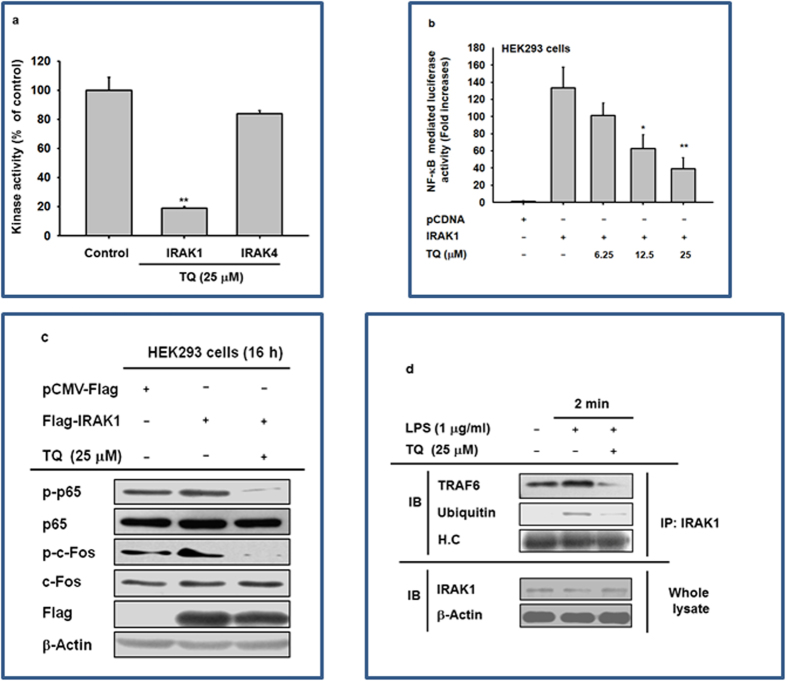
Effect of TQ on the activation of IRAK1. (**a**) Kinase activities of IRAK1 and IRAK4 were determined by a kinase profiler service using purified enzymes and substrate. The vehicle control was set to 100% activity for IRAK1 and IRAK4 enzymes for the purpose of comparison with treated cells. (**b**) Effect of TQ on IRAK1-induced NF-κB activation was measured by a reporter gene assay. The luciferase activity of HEK293 cells transfected with NF-κB-Luc (1 μg/mL) and IRAK1 in the presence or absence of TQ was measured using a luminometer. (**c**) Effect of TQ on the activation of AP-1 and NF-κB upon IRAK1 overexpression was assessed by immunoblot analysis of phosphorylated and total levels of p65 and c-Jun. (**d**) Effect of TQ on the formation of the IRAK1-TRAF6 complex and ubiquitinylation was determined by immunoblot analysis. The blots in c and d were obtained under the same experimental conditions and are shown as cropped blots (original blots with indicated cropping lines are shown in Supplementary Figure S1). The data shown represent mean ± SD of three (**a**) or five (**b**) samples. Results in **c** and **d** are shown for one representative experiment of three. *p < 0.05 and **p < 0.01 compared with control.

Building on our immunoblot data, we next used a kinase activity assay to examine whether TQ suppresses the enzymatic activity of IRAKs. As expected, TQ abolished the activity of IRAK1, but not IRAK4 (Fig. 5a). As an alternative approach to validating IRAK1 as a target of TQ, we developed an NF-κB–driven luciferase reporter assay. A plasmid construct encoding IRAK1 was cotransfected with the NF-κB–driven luciferase plasmid construct to induce luciferase activity in HEK293 cells. As expected, IRAK1 increased NF-κB–driven luciferase activity by 138-fold relative to the vector-only control (Fig. 5b); however, TQ reduced luciferase activation by up to 80% in a dose-dependent manner. Concurrently, TQ reduced the levels of IRAK1-mediated p65 and c-Jun phosphorylation (Fig. 5c). TQ treatment also inhibited the production of ubiquitinated IRAK1 and formation of the complex of TRAF6 with IKAK1 (Fig. 5d).

## Discussion

With this work, we explored the mechanism by which TQ regulates inflammation using both *in vitro* (TLR2/3/4-stimulated primary and cancerous macrophages) and *in vivo* (mouse gastritis and hepatitis models) experimental conditions. We consider TQ to be a promising candidate for medicinal treatments in part because TQ-containing plants are common traditional anti-oxidative and anti-inflammatory remedies with proven efficacy in various disease models, including encephalomyelitis, diabetes, and asthma^27^. The status of TQ as an important anti-inflammatory compound motivated us to identify the molecular mechanism underlying its anti-inflammatory activity.

As initially hypothesized, TQ decreased the secretion of NO and PGE_2_ and downregulated inflammatory gene expression in activated macrophages. TQ also inhibited the expression of multiple genes involved in these processes including IL-6, TNF-α, iNOS, and COX-2 in LPS-, pam3CSK-, and poly (I:C)-stimulated macrophage-like RAW264.7 cells (Figs 1, 2 and 3). Significantly, TQ had these effects without impacting cell viability (Fig. 1d) or normal inflammatory gene expression patterns (Fig. 3a lower panel). Moreover, TQ alleviated the acute inflammatory symptoms triggered by gastric HCl/EtOH (Fig. 2a) and hepatic LPS/D-GalN (Fig. 2b). We conclude that TQ-induced inhibition of NO and PGE_2_ production and downregulation of pro-inflammatory cytokines (IL-6 and TNF-α) and genes (iNOS and COX-2) at least in part mediate the anti-inflammatory activities of TQ in *in vitro* and *in vivo* conditions (Figs 1, 2 and 3). Additionally, it has previously been reported that TQ and *N. sative*, a plant with high TQ content^35^, suppress allergic airway inflammation by inhibiting the expression of COX-2 and production of PGE_2_^36^. We also found that TQ reduced the inflammatory response associated with pancreatic ductal adenocarcinoma by suppressing NF-κB–mediated inflammatory gene expression^37^. Of note, TQ relieves colitis symptoms in mice induced by a 7-day regimen of dextran sodium sulfate (DSS) (3% W/V) added to the drinking water^38^. Therefore, considering our data and previous reports, it is clear that TQ exerts a general anti-inflammatory effect that may provide a clinically useful treatment for various inflammatory symptoms.

Various experimental approaches including immunoblot analysis, luciferase reporter gene assay, enzyme assay, overexpression of target gene, and immunoprecipitation analysis all confirmed that TQ targets IRAK1, which is involved in the activation of both AP-1 and NF-κB. For example, the nuclear levels of NF-κB subunits, p65 and p50, were apparently decreased when cells were treated with TQ (Fig. 3b). Moreover, TQ simultaneously inhibited the induction of both NF-κB– and AP-1–driven luciferase activities that would otherwise be triggered by MyD88 (Fig. 3f, left panel), a major adaptor molecule delivering TLR-driven stimulation to intracellular signaling enzymes in HEK293 cells^39^. TQ also blocked critical signaling events involved in NF-κB activation, including the phosphorylation of AKT, PDK1, and IκBα after LPS exposure (in RAW264.7 cells); the phosphorylation of IκBα and p65 in the gastric tissue of HCl/EtOH-treated mice; and the AP-1–mediated phosphorylation of JNK, p38, MKK4, and MKK3 after LPS treatment in RAW264.7 cells and in LPS/D-GalN–treated liver tissue (Fig. 4). These results strongly imply that several enzymes involved in the induction of the NF-κB and AP-1 pathways are targeted by TQ.

Based on this assumption, we examined the effect of TQ on the activity of IRAKs triggered by LPS treatment. It has been previously reported that complex formation between MyD88 and IRAK1/2/4 activates molecular interactions between TRAF6, TABs, and TAK1, in turn activating both the IKK/IκBα pathway for NF-κB translocation and the MAPK pathway for AP-1 translocation^40^. Additionally, overexpression of IRAK1 increased the phosphorylation levels of p65 and c-Fos (Fig. 5c). Moreover, other compounds that target IRAK1, such as caffeic acid, kalopanaxsaponin B, anthraquinone-2-carboxylic acid, quercetin, and mangiferin, have been shown to exert strong anti-inflammatory effects by blocking the NF-κB and AP-1 pathways^21^,^22^,^23^,^24^,^41^. Interestingly, TQ restored the reduced level of IRAK1 triggered by gastritis and hepatitis in stomach and liver (Fig. 4d) and blocked the LPS-regulated degradation of IRAK1 at 2 min (Fig. 4e). TQ also directly suppressed IRAK1 kinase activity (Fig. 5a). Other consistent findings include the ability of TQ to dose-dependently reduce NF-κB–driven luciferase activation (Fig. 5b) and its downregulation of the phosphorylation of important NF-κB and AP-1 subunits p65 and c-Jun, which is normally mediated by IRAK1 expression (Fig. 5c). Finally, TQ inhibits the ubiquitylation of IRAK1, which is an important step in its degradation pathway^42^, and of the TRAF6 complex, which is a critical part of TAK1 activation^40^ (Fig. 5d). Together, these data show that IRAK1, a crucial link between TLR stimulation and NF-κB/AP-1 activation, is a likely molecular target of TQ that mediates its anti-inflammatory effects. We do not currently have sufficient data to explain precisely how TQ directly suppresses IRAK1, but this will be part of our continuing work investigating the inhibitory mode of action TQ against IRAK1.

**Figure 6:**
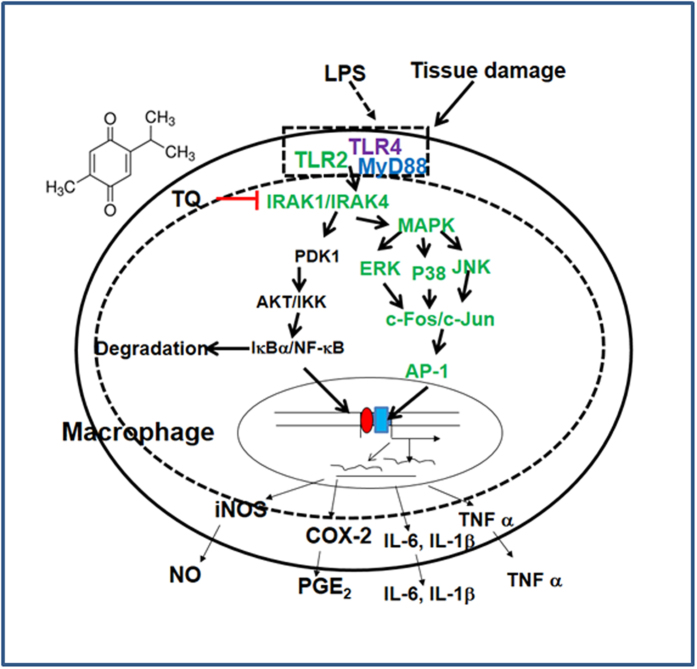
Putative inhibitory pathway of TQ-mediated anti-inflammatory responses.

In conclusion, we found strong evidence that TQ reduces inflammatory responses both *in vitro* and *in vivo* by targeting the enzyme activity and degradation of IRAK1, thereby reducing the activity of downstream NF-κB and AP-1 (Fig. 6). Future experimental trials will focus on additionally validating TQ as a novel anti-inflammatory treatment in preclinical studies, as well as elucidating the key molecular details that underlie the TQ/IRAK1 interaction.

### Materials and methods

Thymoquinone (Purity: 99%), 3-(4,5-dimethylthiazol-2-yl)-2,5-diphenyltetrazolium bromide (MTT), silibinin, *N*^ω^-nitro-L-arginine methyl ester (L-NAME), indomethacine, NSC95395, pam3CSK, Poly (I:C), polyethyleneimine (PEI), and lipopolysaccharide (LPS, *Escherichia coli* 0111:B4) were obtained from Sigma Chemical Co. (St. Louis, MO, USA). Cell culture products such as fetal bovine serum (FBS) and RPMI1640 were purchased from Gibco Products (Grand Island, NY, USA). Cell lines used in these experiments were products of ATCC (Rockville, MD, USA). RAW264.7 cells are a BALB/c-derived murine macrophage cell line (No. TIB-71); U937 cells are a human pleura/pleural effusion monocyte-like cell line (No. CRL-1593.2); and HEK293 cells are a human embryonic kidney cell line (No. CRL-1573). Luciferase constructs with NF-κB and AP-1 binding promoter sites were used as previously reported^43^. All other chemicals were products of Sigma. Catalog numbers for phospho-specific (P) or total (T) antibodies against the listed proteins were as follows: c-Fos (P: 5348/T: SC52), p50 (T: 12540), p65 (P: 3039/T: 8242), IκBα (P: 9246/T: 9242), IκB kinase (IKK)α/β (P: 2697/T: 2682), Akt (Ser 473) (P: 4058/T: 9272), c-Jun-N-terminal kinase (JNK) (P: 9255/T: 4672), extracellular signal-regulated kinase (ERK) (P: 9101/T: 4696), p38 (P: 4631/T: 9212), mitogen-activated protein kinase (MAPK) kinase (MKK3/6) (P: 9236/T: 9232), MKK4 (P: 9151/T: 9152), IRAK1 (T: 4504), IRAK4 (T: 4363), Flag (T: 2368), TRAF6 (T: 8028), ubiquitin (T: 3933), lamin A/C (T: 4777), and β-actin (T: 4967). Antibodies were purchased from Cell Signaling Technology (Beverly, MA, USA) and Santa Cruz Biotechnology (Santa Cruz, CA, USA).

### Cell cultures

RAW264.7 and U937 cells were maintained in RPMI1640, and HEK293 cells were cultured in DMEM. Each medium was supplemented with heat-activated FBS (10%), glutamine, penicillin, and streptomycin, and cells were cultured at 37 °C under 5% CO_2_. Before TQ treatment, U937 cells were differentiated into macrophages by incubation with PMA (20 nM) for 12 h, as previously reported^44^. The differentiated cells were maintained at 37 °C under 5% CO_2_.

### Preparation of peritoneal macrophages

Peritoneal macrophages were prepared from C57BL/6 male mice by injection of sterile thioglycollate broth (4%, Difco Laboratories, Detroit, MI, USA), as reported previously^45^. After washing the cells with FBS (2%)-containing RPMI1640 medium, the peritoneal macrophages (1 × 10^6^ cells/mL) were seeded on 10-mm^2^ dishes at 37 °C for 4 h.

### Drug treatment

TQ was dissolved in 100% DMSO to prepare a 100 mM stock solution and additionally diluted with culture media for *in vitro* experiments. For *in vivo* treatment, TQ was resuspended in 1% Na CMC at concentrations of 5 and 25 mg/kg.

### NO and PGE_2_ production

Pre-incubated RAW264.7 cells (1 × 10^6^ cells/mL) were exposed with TQ (6.25–25 μM) or control drugs (L-NAME or indomethacine) for 30 min and continuously stimulated with LPS (1 μg/mL) for 24 h. The secreted levels of NO and PGE_2_ were measured by Griess assay and enzyme immunoassays (EIAs), as previously reported^8^.

### Cell viability assay

The cell viability of RAW264.7 cells, peritoneal macrophages, and HEK293 cells during TQ exposure for 24 h was determined by a conventional MTT assay, as previously described^46^.

### Animals

Male C57BL/6 and ICR mice (6–8 weeks old, 17–21 g) obtained from Daehan Biolink (Chungbuk, Korea) were housed under conditions of a 12-h light/dark cycle (lights on at 6 am). All experiments were performed according to guidelines of the National Institute of Health for the Care and Use of Laboratory Animals (NIH Publication 80–23, revised in 1996) and with approval of the Institutional Animal Care and Use Committee at Sungkyunkwan University (Suwon, Korea; approval ID: SKKUBBI 12-6-1).

### *In vivo* EtOH/HCl-induced gastritis mouse model

Gastritis symptoms in the stomach were triggered by oral administration of EtOH/HCl in accordance with a previously described method^47^. Fasted ICR mice were orally treated with either TQ (5 and 25 mg/kg) or ranitidine (40 mg/kg) 2 times a day for 3 days. Thirty minutes after the final administration, 400 μL of 60% ethanol in 150 mM HCl was orally administered. Mice were anaesthetized and sacrificed with an overdose of ether 1 h after administration of the necrotizing agents. After opening the stomach, we measured the inflamed area (mm^2^) with gastric ulcer lesions and quantified lesions using custom ImageJ-based software, as previously reported^47^.

### *In vivo* LPS/D-GalN–induced hepatitis mouse model

A mouse model of experimental liver inflammation was induced by injection of LPS according to a previously published method^44^. Briefly, C57BL/6 mice (5 weeks old) were orally treated with TQ (25 mg/kg) or silibinin (100 mg/kg) once a day for 6 days using crop needles. One hour after the final treatment with TQ, LPS (10 μg/kg) and D-GalN (1 g/kg) were injected intraperitoneally. After 6 h, the animals were anesthetized by urethane overdose, and blood was collected by cardiac puncture. Serum was obtained by centrifugation of blood at 3,000 rpm for 15 min. Serum levels of alanine aminotransferase (ALT) and aspartate aminotransferase (AST) were measured using a Roche Modular spectrophotometric autoanalyzer.

### Measurement of mRNA by reverse transcriptase-polymerase chain reaction (RT-PCR) and real-time PCR

To determine cytokine mRNA expression levels, RAW264.7 cells were pre-treated with TQ (12.5–25 μM) for 30 min and U937 cells for 3 h and then incubated with LPS (1 μg/mL for RAW264.7 cells and 10 μg/mL for U937 cells) for an additional 6 h (RAW264.7 cells) or 12 h (U937 cells). Total RNA was extracted using TRIzol Reagent (Gibco) in accordance with the manufacturer’s protocol and kept at −70 °C for later use. Quantitative and semi-quantitative RT-PCR reactions were performed as previously described^48^. Primers from Bioneer (Seoul, Korea) are listed in Table 1.

### Plasmid transfection and luciferase reporter gene activity assay

For reporter gene assays, plasmids (1 μg/mL each) encoding the luciferase gene with promoters containing AP-1 or NF-κB binding sites were transfected into RAW264.7 and HEK293 cells (1 × 10^6^ cells/mL) under cotransfection conditions with IRAK1, MyD88, or LPS (1 μg/mL) by a polyethylenimine (PEI) method. After stabilization for 12 h, the transfected cells were treated with TQ for 12 h. Luciferase activity was measured using the Luciferase Assay System (Promega, Madison, WI, USA), as previously reported^49^. To evaluate total and phosphorylated c-Jun and p65, we transfected IRAK1 into HEK293 cells (1 × 10^7^ cells/mL) for 48 h. The cells were additionally treated with TQ for the last 12 h. The levels of total and phosphorylated c-Jun, p65, and β-actin were determined from whole-cell lysates of IRAK1-overexpressing cells by immunoblot analysis.

### Extraction of total lysates/nuclear fraction and western blotting

Total lysates of peritoneal macrophages (5 × 10^6^ cells/mL), HEK293 cells (5 × 10^6^ cells/mL), PMA-differentiated U937 cells (2.5 × 10^6 ^cells/mL), RAW264.7 cells (5 × 10^6^ cells/mL), stomachs, and livers were prepared according to a previously reported method^50^. Nuclear fractions of RAW264.7 cells were prepared from RAW264.7 cells (5 × 10^6^ cells/mL) by a three-step procedure, as reported previously^51^. The whole lysates and nuclear fractions were subsequently used in immunoblot analyses to analyze the phosphorylated and total levels of transcription factors (c-Fos, p65, and p50) and signaling proteins (IκBα, IKK, AKT, JNK, p38, ERK, MKK3/6, MKK4, IRAK1, IRAK4, and TRAF6), as previously described^49^. Immunoprecipitation analysis was carried out with whole-cell lysates^49^, and the isolated proteins were visualized by immunoblot analysis.

### IRAK1/4 kinase assay

To assess the inhibitory action of TQ compound on the activity of purified IRAK1/4 enzymes, we used a kinase profiler service from Millipore (Billerica, MA, USA), involving a radiometric assay measuring the radioactivity of IRAK1/4 substrates incorporated by IRAK1/4 52. Radioactivity of IRAK1/4 substrates was analyzed by scintillation counter.

### Histopathology

Histopathological examinations were performed as previously described^53^. Stomach and liver tissues were stained with hematoxylin and eosin and examined for signs of tissue injury under a photomicroscope.

### Statistical analysis

All data in this study are presented as mean ± standard deviation (SD) calculated from three samples (enzyme assay), five samples (*in vitro* experiments), or seven mice (*in vivo* experiments). For statistical comparisons, we analyzed all values using ANOVA/Scheffe’s post hoc test as well as the Kruskal–Wallis/Mann–Whitney tests. A *P*-value < 0.05 was accepted as statistically significant. Statistical evaluation was carried out with SPSS software (SPSS Inc., Chicago, IL, USA). Similar experimental data were obtained from an additional independent set of *in vitro* and *in vivo* experiments performed under the same conditions.

## Additional Information

**How to cite this article:** Hossen, M. J. *et al*. Thymoquinone: An IRAK1 inhibitor with *in vivo* and *in vitro* anti-inflammatory activities. *Sci. Rep.* **7**, 42995; doi: 10.1038/srep42995 (2017).

**Publisher's note:** Springer Nature remains neutral with regard to jurisdictional claims in published maps and institutional affiliations.

## Supplementary Material

Supplementary Figure 1

## Figures and Tables

**Table 1:** Sequences of RT-PCR and real-time PCR primers used in this study.

Name	Sequence (5′ to 3′)	
RT-PCR (murine genes)		
TNF-α	F	TTGACCTCAGCGCTGAGTTG
	R	CCTGTAGCCCACGTCGTAGC
iNOS	F	CCCTTCCGAAGTTTCTGGCAGCAG
	R	GGCTGTCAGAGCCTCGTGGCTTTGG
IL-6	F	GGAAATCGTGGAAATGAG
	R	GCTTAGGCATAACGCACT
IL-1β	F	CAGGATGAGGACATGAGCAC
	R	CTCTGCAGACTCAAACTCCA
GAPDH	F	CAATGAATACGGCTACAGCAAC
	R	AGGGAGATGCTCAGTGTTGG
Real-time PCR (murine genes)		
iNOS	F	GGAGCCTTTAGACCTCAACAGA
	R	TGAACGAGGAGGGTGGTG
COX-2	F	CACTACATCCTGACCCACTT
	R	ATGCTCCTGCTTGAGTATGT
TNF-α	F	TGCCTATGTCTCAGCCTCTT
	R	GAGGCCATTTGGGAACTTCT
IL-1β	F	TAGAGCTGCTGGCCTTGTTA
	R	ACCTGTAAAGGCTTCTCGGA
IL-6	F	AAGCCAGAGCTGTGCAGATGAGTA
	R	CTTGGTCACCGACGTCCTGT
GAPDH	F	CAATGAATACGGCTACAGCAAC
	R	AGGGAGATGCTCAGTGTTGG
Real-time PCR (human genes)		
COX-2	F	ACTGTACGGGGTTTGTGACTAG
	R	ACTGTACGGGGTTTGTGACTAG
TNF-α	F	GAAAGCATGATCCGGGACGTG
	R	GATGGCAGAGAGGAGGTTGAC
IL-1β	F	CCGACCACCACTACAGCAAG
	R	GGGCAGGGAACCAGCATCTT
IL-6	F	AAGCCAGAGCTGTGCAGATGAGTA
	R	CTTGGTCACCGACGTCCTGT
GAPDH	F	TGGAAGGACTCATGACCACA
	R	AGGGGTCTACATGGCAACTG
