# Supplementary material for: Thymoquinone: An IRAK1 inhibitor with in vivo and in vitro anti-inflammatory activities
Source: Sci Rep. 2017 Feb 20;7:42995. doi: 10.1038/srep42995 (PMC5316937; doi:10.1038/srep42995)
Supplement: Supplementary Figure 1 [file srep42995-s1.pdf]

Supplementary Information

Thymoquinone: an IRAK1 inhibitor with *in vivo* and *in vitro* anti-inflammatory activities

Muhammad Jahangir Hossen[1,2,a], Woo Seok Yang[1,a], Daewon Kim[3], Adithan Aravinthan[3]

Jong-Hoon Kim[3,**]Jae Youl Cho[1,*]

[1]Department of Genetic Engineering, Sungkyunkwan University, Suwon 16419, Republic of Korea. [2]Department of Animal Science, Patuakhali Science and Technology University, Dumki, Patuakhali 8602, Bangladesh. [3]Laboratory of Bio-informatics, Department of Multimedia Engineering, Dankook University, Cheonan 31116, Republic of Korea. [a]These authors contributed equally to this work. Correspondence and requests for materials should be addressed to J.Y.C. (jaecho@skku.edu) or D.K. (dr_dwkim@dankook.ac.kr)

1. The cropping lines of gels/blots

Selected data were shown in our manuscript and the PVDF membranes for Western Blot were cut into strips to minimize the amount of antibodies that are necessary for analysis.
